# Consistency of Violence: Implications for Effective Inpatient Psychiatric Care

**DOI:** 10.1007/s10488-023-01251-4

**Published:** 2023-03-14

**Authors:** Lucy McIvor, James Payne-Gill, Helen Winter, Clair Pollard, Alison Beck

**Affiliations:** Corporate Psychology and Psychotherapy, South London and Maudsley NHS Foundation Trust, Maudsley Hospital, Denmark Hill, London, SE5 8AZ UK

**Keywords:** Violence, Aggression, Inpatients, Mental Health Services, Psychiatry

## Abstract

Instances of violence and aggression in acute psychiatric settings are common and highly distressing for service users and staff. They also incur financial costs. This study aimed to identify the proportion of service users at risk of consistent violence/aggression enactment. It also aimed to analyse associated service use to explore the potential need for specialised, targeted approaches. Five years’ worth of data were extracted from 2016 to 2020 on inpatient stays across South London and Maudsley NHS Foundation Trust (SLaM) acute adult wards and Psychiatric Intensive Care Units (PICUs). Service users were divided into cohorts based on relative number of violent/agressive incidents enacted. Differences in frequency of acute service use during the period 1^st^ January-31^st^ December 2020 were analysed. In total, 2524 service users had at least one inpatient stay during 2020. 679 were recorded as having enacted at least one incident of violence or aggression. Just 4% of all service users accounted for 50% of all violence/aggression enactment. Results further showed strong evidence of group differences between violence cohorts in the following domains: internal transfers, occupied bed days, admissions and Place of Safety (PoS) referrals. There was weaker evidence for group differences in referrals to Home Treatment teams (HTTs) and Psychiatric Liaison Teams. A small proportion of service users disproportionately account for the majority of violent and aggressive incidents and higher levels of violence and aggression are associated with more acute service use. The provision of targeted, personalised interventions for this cohort may reduce the enactment of violence and aggression, leading to improved quality life and a reduction in financial expenditure.

## Introduction

Instances of violence and aggression are highly distressing for staff and service users (Serper et al., 2005). For those who enact violence and aggression, immediate consequences include restraint, seclusion and forced medication, which may be experienced as traumatic (Iozzino et al., 2015). For service users targeted by violence and aggression, consequences include anxiety, traumatisation and increased mental distress (Renwick et al., 2016). For healthcare professionals, frequently witnessing or being targeted by violence and aggression can lead to burnout, which aside from being distressing to experience may have a detrimental impact on healthcare professionals’ ability to provide compassionate care (Foster et al., 2007). An improved understanding of violence and aggression is therefore key to developing preventative measures to alleviate distress caused (McIvor et al., 2022). There is variation in the reported proportion of inpatients enacting violence and aggression on acute psychiatric wards, with study estimates ranging between 17–40% (Iozzino et al., 2015; Lockertsen et al., 2020). In the United Kingdom (UK) specifically, predictions lie on the upper bound of this estimate (Renwick et al., 2016).

In addition to causing human distress, the occurrence of violence and aggression also incurs financial costs. Arguably, current policy does not recognise the prevalence of violence and aggression within acute settings, resulting in guidelines that do not sufficiently elaborate working practices in relation to service users who enact violence and aggression (Ferracuti et al., 2022; Watt et al., 2018). In psychiatric wards in England, the estimated direct annual costs of assaults, verbal abuse and damage to objects is £20.5 million per year (2013/2014 prices, NICE, 2015). These estimates are based on staff time taken and resources directly used to manage the incidents and does not include secondary costs associated with service users who enact violence and aggression, e.g. an increased number of occupied bed days (OBDs) and admissions (Cornaggia et al., 2011; Dack et al., 2013; Grassi et al., 2006). These service users may also have an increased number of ward transfers during hospital stays, be it to an acute ward or a specialist forensic care setting. Both result in disruption to care and higher costs. Interestingly, violence and aggression throughout the life span is protective of enacting violence and aggression in hospital in a forensic context. In explanation it may be that there are clearer guidelines for identifying and managing service users with a serious violence history in forensic compared to acute settings (Grevatt et al., 2004; Smith et al., 2020).

In the UK, a person deemed to have serious mental health issues may be admitted voluntarily to an acute psychiatric ward if they agree to an admission. Alternatively, a person may be detained in hospital under the Mental Health Act (1983) (often known as ‘being sectioned’) if they do not consent to admission. The decision to detain someone must be made by a medical doctor and an approved mental health professional (Royal College of Psychiatrists, 2022). As an individual’s risk to self and others is given primacy in the assessment of need for admission, an inpatient cohort self-selects for potential instances of violence/aggression enactment. Though often necessary, the decision to detain someone is ultimately subjective and depends on structural influences (e.g. level of pressure experienced by services) and the therapeutic relationship and interaction between doctor and service user (McIvor et al., 2022). The two most common types of section for those detained on acute psychiatry wards are ‘admission for assessment’ (lasting a maximum of 28 days) and ‘admission for treatment,’ (lasting for a maximum of six months before renewal is needed). (Mental Health Law, 2021).

In an acute psychiatric setting, the most replicated and robust variable correlated with the enactment violence and aggression is a past history of violence and aggression (Cornaggia et al., 2011; Dack et al., 2013; Grassi et al., 2006; Meehan et al., 2017; Mellesdal, 2003; Renwick et al., 2016; Rueve & Welton, 2008; Soliman & Reza, 2001; Steinert, 2002). Of note, there is evidence that a small minority of service users account for the majority of violent/aggressive incidents. Certain research has found that 22% of service users involved in three or more incidents accounted for 53% of all instances of violence (Convit et al., 1990), supported by further research reporting that just 13% of service users enacting instances of aggression accounted for nearly 50% of aggressive incidents on inpatient wards (Mellesdal, 2003). Both findings are further supported by a recent meta-analysis exploring instances of violence and aggression across a range of settings. Results showed that on average, 36.6% of service users enacting instances of violence were involved in and disproportionately accounted for the majority of repeat violent incidents in acute psychiatric settings (Bowers et al., 2011).

## Summary

Instances of violence and aggression can have a distressing impact on service users and staff, as well as leading to increased service use and financial expenditure. In this paper we aimed to explore differences in acute service use across service users enacting different frequencies of violent and aggressive incidents within South London and Maudsley NHS Foundation Trust (SLaM). The primary aim of this was to explore the potential need for a specialised approach when working with the small group of service users disproportionately accounting for the enactment of the majority of violent and aggressive incidents. As such, the emphasis of this paper is exploration, and conjecture on the impact of a specialised approach is speculative. We aimed to address gaps in previous research through identifying dynamic changes in the enactment of violence and aggression over a five-year period, in addition to providing information on forecasted expenditure.

## Method

### Data Collection: Incident and Service Use Data

Data on violent and aggressive incidents were extracted from Datix, the Trust’s incident recording system, whilst service use data was extracted from The Cube, an SQL server that stores service use data. Datix and Cube data were matched using a service user’s Trust-ID. Overall, we were able to match 80.29% of data. In explanation, a total of 2258 incidents were recorded as being enacted by 679 service users. We are able to provide detailed information for 1858 of these incidents. The data availability per violence cohort can be found in Appendix F.

### Incident Data: Violence Cohort Grouping and Consistency of Violence Enactment

Five years’ worth of data were extracted from 1st January 2016 to 31st December 2020 on service users recorded as an enactor of violent/aggressive incidents and service users who had an inpatient stay across SLaM’s acute adult wards and Psychiatric Intensive Care Units (PICUs).

The purpose of the 2020 incident data was to divide service users into violence cohorts (‘low’, ‘medium’ or ‘high’) based on the relative number of violent/aggressive enacted across the year. The purpose of the 2016–2019 data was to identify the cohort of service users who had been in the most violent cohort in at least one year prior 2020, to form the ‘high and consistent violence cohort’ and to explore the consistency of violence/aggression enactment over time.

We identified 2524 service users with at least one inpatient stay during 2020. Of these, 679 were recorded as having enacted at least one incident of violence or aggression during the year. The maximum number of incidents a service user enacted was 49. The cumulative distribution of violent or aggressive incidents across the year is skewed and showed that 104 service users (which amounts to 15% of the service users involved in at least one violent incident but just 4% of all service users with an acute adult ward or PICU stay) accounted for 50% of violence/aggression, while 304 service users accounted for 80% of violence/aggression.

Based on these findings, we grouped service users into five cohorts:

1. ‘No violence’ cohort: Service users with an inpatient stay but with no violent/aggressive incidents recorded (N = 1,845)
2. ‘Low violence’ cohort: Service users comprising the 80–100% section of the distribution (N = 376)
3. ‘Medium violence’ cohort: Service users comprising the 50–80% section of the distribution (N = 199)
4. ‘High violence’ cohort: Service users comprising the 0–50% section of the distribution (N = 74)
5. ‘High and consistent violence’ cohort: This cohort comprised of service users in the ‘high violence’ cohort making up the 0–50% section of the distribution in 2020, who also comprised this part of the distribution in previous years (2016, 2017, 2018 or 2019) (N = 30)

### Service Use Data

Differences in frequency of acute service use during 1^st^ Janaury-31^st^ December 2020 were then analysed. Acute service use was defined as accepted referrals to Psychiatric Liaison Teams, HTTs and Place of Safety (PoS), admissions to inpatient wards, internal transfers between wards, and total OBDs across the year.

### Violence and Aggression Definition

Within this study, we have defined ‘the enactment of violence and aggression’ via the categories shown in Table 1. These are predefined Datix categories, each of which has several subcategories (Appendix A-E). When an incident occurs, clinical staff members on acute wards are responsible for recording the incident and assigning the incident to a Datix category and subcategory.

**Table 1:** Summary of violent/aggressive incident types enacted for individual (low, medium, high, high and consistent) violence cohorts and all violence cohorts combined (N = 1,858)

	Assault by patient including alleged	Challenging Behaviour	Harassment by patient including alleged	Sexual assault by patient including alleged
% of total violence				
All cohorts combined	51.99	43.92	2.74	1.35
Low violence cohort	43.51	51.35	2.70	2.43
Medium violence cohort	51.35	45.59	2.16	0.90
High violence cohort	55.52	40.80	2.17	1.51
High and consistent violence cohort	56.12	38.51	4.78	0.60

The variable ‘assault by patient including alleged’ includes actual or threatened assaults by service users on staff, visitors, or other service users.

The variable ‘challenging behaviour’ includes actual and alleged inappropriate sexual behaviour, aggression resulting in accidental injury, throwing objects aggressively and verbal assault. This variable also includes violence/aggression classed as ‘other/not stated’.

The variable ‘harassment by patient including alleged’ includes alleged, sexual, racial or ‘other’ harassment by service users on staff, visitors or other service users. The variable ‘sexual assault by patient including alleged’ includes actual or threatened sexual assaults by service users on staff, visitors or other service users.

Whilst the definition encompasses a wide range of actual or threatened behaviours, for all violence cohorts combined, over 50% of incidents in every category have a severity rating of moderate and above (Appendix A). This may indicate that broadly, the enactment of violence and aggression has a tangible impact regardless of category. The most commonly reported category varied between groups. For the low violence group ‘challenging behaviour’ was the most common whilst for the medium, high, and high and consistent violence groups, ‘assault by patient including alleged’ was the most common (Table 1).

### Data Reliability

With regards to reliability, the service use data used in this study (e.g. number of admissions, transfers, OBDs etc.) is objective and accurate. The incident data collected consists of entries made by healthcare staff in response to violent and aggressive incidents on the ward. The recording of this data is therefore more subjective, in that there are a series of decisions involved that ultimately rest with the staff member, including whether to record an incident, how to categorize the incident, and what severity rating to class an incident. There is therefore likely to be variation between individual staff members as to how incidents are recorded. We believe this data is still valuable and note that any exploration of violence and aggression that is not purely theoretical (e.g. exploring actual incidents that have occurred) is likely to incur an element of human bias.

### Statistical Analysis

Kruskal–Wallis tests were conducted to analyse violence cohort differences in acute service use in 2020. Kruskal–Wallis tests identify overall cohort differences but do not show which individual cohorts are statistically different from one another. Therefore, in addition to the group tests, we conducted pairwise Wilcoxon-Signed rank tests to identify which cohorts displayed statistically significant differences from one another.

### Ethical Approval

Approval was received by the SLaM Ethical Approval Committee for clinical audits, service evaluations and other quality improvement projects (ID PPF20201218) and has therefore been performed in line with the principles of the Declaration of Helsinki. Data was stored in accordance with the Trust’s information governance procedures and was anonymised at the earliest possible opportunity during the analysis process in order to protect confidentiality.

## Results

### Differences in Incident Rate and Acute Service use between Violence Cohorts

As anticipated, a Kruskal–Wallis test showed highly significant group differences in incident rate between violence cohorts. Kruskal–Wallis tests also showed highly significant group differences between violence cohorts in the following outcomes: internal transfers, OBDs, admissions and PoS referrals. There were also significant differences in referrals to HTTs between violence cohorts (Table 2).

**Table 2:** Descriptive and Kruskal–Wallis statistics for rate of incidents and each service use metric for individual violence cohorts and all violence cohorts combined

	No violence (N = 1845)	Low violence (N = 376)	Medium violence (N = 199)	High violence (N = 74)	High and consistent violence (N = 30)	Overall (N = 2524)	Kruskal–Wallis statistic	p-value
Rate of incidents								
Mean (SD)	0(0)	1.20(0.40)	3.40(1.09)	9.65(5.89)	13.83[7.94]	0.89 (2.68)	2511	< 0.001***
Median [Min, Max]	0[0,0]	1.00[1.0,2.0]	3.00[2.0,6.0]	8.00 [6.0, 49.0]	11.00[6.0, 41.0]	0[0,49.0]		
Transfers								
Mean (SD)	0.886 (1.51)	1.82 (2.38)	3.15 (3.51)	4.01 (3.30)	10.4 (6.09)	1.41 (2.42)	396	< 0.001***
Median [Min, Max]	0 [0, 17.0]	1.00 [0, 19.0]	2.00 [0, 28.0]	3.00 [0, 17.0]	9.50 [3.00, 27.0]	1.00 [0, 28.0]		
Occupied Bed Days								
Mean (SD)	33.4 (38.4)	57.8 (52.0)	77.6 (59.1)	117 (81.5)	176 (92.9)	44.6 (51.0)	432	< 0.001***
Median [Min, Max]	22.0 [1.00, 354]	45.0 [1.00, 357]	60.0 [3.00, 363]	102 [3.00, 366]	194 [21.0, 314]	28.0 [1.00, 366]		
Admissions								
Mean (SD)	1.77 (1.51)	2.32 (2.19)	2.54 (2.16)	2.39 (1.75)	5.10 (3.35)	1.97 (1.78)	127	< 0.001***
Median [Min, Max]	1.00 [0, 27.0]	2.00 [0, 21.0]	2.00 [0, 14.0]	2.00 [0, 8.00]	4.00 [1.00, 16.0]	1.00 [0, 27.0]		
Psychiatric Liaison Team								
Mean (SD)	2.73 (5.07)	2.70 (4.76)	2.99 (4.75)	2.72 (3.75)	4.97 (5.48)	2.77 (4.97)	9.26	0.099
Median [Min, Max]	1.00 [0, 66.0]	1.00 [0, 56.0]	1.00 [0, 29.0]	2.00 [0, 20.0]	2.50 [0, 20.0]	1.00 [0, 66.0]		
Home Treatment Team								
Mean (SD)	1.41 (1.75)	1.43 (1.79)	1.74 (2.04)	1.88 (1.94)	2.33 (2.75)	1.46 (1.81)	11.6	0.0403*
Median [Min, Max]	1.00 [0, 17.0]	1.00 [0, 13.0]	1.00 [0, 12.0]	1.00 [0, 8.00]	1.00 [0, 9.00]	1.00 [0, 17.0]		
Place of Safety								
Mean (SD)	0.357 (0.815)	0.582 (1.15)	0.613 (1.09)	0.581 (0.936)	2.23 (2.69)	0.439 (0.966)	87.7	< 0.001***
Median [Min, Max]	0 [0, 10.0]	0 [0, 15.0]	0 [0, 9.00]	0 [0, 5.00]	1.00 [0, 11.0]	0 [0, 15.0]		

^*^p < .05, **p < .01, ***p < .001

Table 3 shows which cohorts displayed statistically significant differences from one another.

**Table 3:** Post-hoc (Wilcoxon-Signed rank) tests conducted to investigate differences between service use metrics for low, medium, high and high and consistent violence cohorts

Comparison Cohort									
Outcome	Cohort	No violence (W)	No violence	Low violence (W)	Low violence	Medium violence (W)	Medium violence	High violence (W)	High violence
Transfers	Low violence	− 12.63	p < 0.001***						
	Medium violence	− 17.86	p < 0.001***	7.57	p < 0.001***				
	High violence	− 16.47	p < 0.001***	9.88	p < 0.001***	3.86	0.006**		
	High and consistent violence	− 14.16	p < 0.001***	12.02	p < 0.001***	9.95	p < 0.001***	8.323	p < 0.001***
OBDs	Low violence	− 16.23	p < 0.001***						
	Medium violence	− 18.96	p < 0.001***	6.85	p < 0.001***				
	High violence	− 16.02	p < 0.001***	10.47	p < 0.001***	6.05	p < 0.001***		
	High and consistent violence	− 11.35	p < 0.001***	9.23	p < 0.001***	7.43	p < 0.001***	4.14	0.003**
Admissions	Low violence	− 7.82	p < 0.001***						
	Medium violence	− 8.65	p < 0.001***	2.19	0.152				
	High violence	− 5.59	p < 0.001***	1.47	0.332	− 0.11	0.941		
	High and consistent violence	− 11.66	p < 0.001***	8.80	p < 0.001***	7.55	p < 0.001***	7.12	p < 0.001***
HTT	Low violence	− 0.55	0.773						
	Medium violence	-3.34	0.094	2.55	0.179				
	High violence	− 3.32	0.094	2.90	0.136	1.03	0.665		
	High and consistent violence	− 1.69	0.463	1.48	0.492	0.63	0.773	0.02	0.994
Place of Safety	Low violence	-7.82	p < 0.001***						
	Medium violence	− 6.28	p < 0.001***	0.30	0.967				
	High violence	− 3.92	0.008**	0.15	0.967	− 0.06	0.967		
	High and consistent violence	− 9.97	p < 0.001***	6.89	p < 0.001***	6.38	p < 0.001***	5.63	p < 0.001***

^*^ p < .05, **p < .01, ***p < .001

The median number of transfers and OBDs increased significantly with each ‘step-up’ in violence, from the ‘no violence’ cohort to the ‘high and consistent violence’ cohort. The median number of admissions for the ‘high and consistent violence’ cohort was statistically higher than all other cohorts, while the median number of admissions for the ‘no violence’ cohort was statistically lower than all other cohorts. There were no significant differences in median number of admissions between the low, medium and high violence cohorts. The same pattern emerged for PoS referrals. There were no significant differences between groups in terms of accepted referrals to HTTs (Table 3).

### Consistency of Violence Enactment over Time (2016 – 2020)

In addition to analysing whether the cohorts involved in the most incidents of violence or aggression also had higher incidences of acute service use, we wished to understand how these cohorts evolved over time. 30 out of the 104 service users comprising the 0–50% of the violence distribution in 2020 additionally comprised the 0–50% of the violence distribution in the years 2016, 2017, 2018 or 2019 (which was used to define the ‘high and consistent violence’ cohort analysed above). Of these 30, 20 were in the ‘high violence’ cohort in one previous year, six were in the ‘high violence’ cohort for two previous years, and four were in the ‘high violence’ cohort for three previous years. Prior to 2020, 47 of the 104 service users had no recorded incidents of violence.

## Discussion

Data shows that of the 2524 service users with at least one inpatient stay during 2020, 679 individuals (approximately one quarter) were recorded as having enacted at least one incident of violence or aggression during the year. This is a relatively high proportion of service users, considering extensive evidence to suggest that those with mental health difficulties are no more likely to enact violence/aggression as compared to the general population (Glied & Frank, 2014). Despite this, of those with mental health difficulties, service users involuntarily admitted or hospitalized accrue the highest rates of reported violence/aggression enactment (20–44%), a figure which broadly concurs with the findings of our study (Varshney et al., 2016).

One interpretation of this high prevalence of violence/aggression is that service users enacting violence or displaying aggressive behaviours are more likely to be hospitalised, as an individual’s risk to self and others is given primacy in the assessment of need for admission. In the UK, as we continue to move towards community-based models of care, Community Mental Health Teams (CMHTs) and Home Treatment Teams (HTTs) work as filters, such that inpatient care is reserved for those who are seriously distressed (NHS, 2019; Royal College of Psychiatrists, 2007).

Alternatively, and equally as plausibly, service users’ experience of hospitalization may contribute to the enactment of violence/aggression. Beyond service user characteristics, a wealth of research has identified a range of environmental factors as contributing the enactment of violence on acute psychiatric wards, including overcrowding (Daffern et al., 2004; Virtanen et al., 2011), poorly designed wards (Ulrich et al., 2018) and staff related influences, such as negative staff morale, staff-staff conflict, negative staff change and pressured staff (Papadopoulous et al., 2012). Indeed, on many occasions the enactment of violence is in itself a reaction to staff intervention (Nawaz et al., 2021). Policy and intervention to reduce violence on inpatient wards should therefore consider service users’ experience of hospitalization, the inpatient environment and the interactions between service users and staff.

The results further show that the distribution of incidents of violence and aggression across service users is heavily skewed. Just 4% of service users (104 out of 2524) with an acute adult ward or PICU stay in 2020 accounted for 50% of all instances of violence and aggression on these wards across the year. Furthermore, this cohort is not transient. 30 of the 104 service users had additionally been in the high violence cohort in at least one of the previous four years. These findings support previous notions that a small proportion of service users disproportionately account for the majority of violent incidents on acute inpatient psychiatric wards (Bowers et al., 2011; Mellesdal, 2003).

Prior to 2020, 47 out of 104 service users had no recorded incidents of violence or aggression. For this cohort newly involved in violence/aggression, we must expand our line of inquiry to question why this may be. One explanation is that service users newly involved in violence have transitioned from child and adolescent mental health services (CAMHS) to adult services, or that they have relocated from another area. A further (and less explored explanation) is one which accounts for structural inequality, changing sociopolitical context, and the consistent othering of certain groups of people (the target of which changes but the presence of which is constant). We believe such an explanation can be summarised through the question ‘how do social, political, and cultural changes manifest in individual behaviour, and how can healthcare systems adapt to support those affected?’ Clinicians must ask these questions and listen carefully to the answer. Individual expressions of distress through violence and aggression cannot, and should not, be separated from wider context. It should further be noted that the COVID-19 pandemic is likely to have contributed to an increase in violent and aggressive incidents in 2020. Research shows that during the lockdown period 23^rd^ March-15^th^ June, there was a 35% increase in the rate of violence and aggressive incidents on inpatient mental health wards within SLaM. This indicates that restrictions to life on the wards had a profound effect on the wellbeing of service users (Payne-Gill et al., 2021).

Our results further demonstrate that higher levels of violence and aggression are associated with significantly higher acute service use (internal transfers, OBDs, internal transfers and PoS referrals). This indicates that interventions aiming to assist service users enacting numerous violent incidents should also account for patterns of service use. The average cost per bed per annum in a South London acute psychiatric ward is £147 000, which translates to approximately £402.73 per OBD (South London Mental Health & Community Partnership., 2018). The 30 service users in the ‘high and consistent violence’ cohort in our study stayed, on average, for 142.6 days longer than service users in the ‘no violence’ cohort in 2020. This amounts to 4,278 excess OBDs for this cohort, at the cost of £1.72 million. Four service users in the ‘high and consistent violence’ 2020 cohort in our study were additionally in the ‘high violence’ cohort for three previous years. Following the assumptions above, this amounts to 570.4 excess OBDS per year for these four individuals, which over four years amounts to 2,281.6 excess OBDs, at the cost of £918, 868. We suggest this presents a strong economic argument for focused work to support the very small number of service users enacting the majority of inpatient violence.

The most common category of violence/aggression enactment for all violence groups was either ‘assault by patient including alleged’, or ‘challenging behaviour’, making up at least 90% of all violent/aggressive incidents for each group. The proportion of incidents classed as ‘moderate’ or above was highest for the ‘high and consistent violence’ group within both of these categories. Therefore, in addition to the increased patterns of service use for this cohort, there is also evidence that the incidents enacted tend to be more severe, further supporting the need for a specialised approach. In the absence of such an approach thus far, it is possible that these service users might be better placed in forensic services, where their behaviour may be managed more easily. The downsides of this approach are that from a financial perspective, forensic services are more expensive (Hare Duke et al., 2018). From a human perspective, individuals who have enacted violence and aggression will additionally have to reckon with the knowledge of having entered a forensic context, which may add to the already often corrosive shame that comes from spending time on an inpatient hospital ward and behaving in a violent/aggressive manner when in distress.

From a theoretical perspective, it is interesting that higher levels of violence/aggression enactment were significantly associated with more acute service use, primarily because different services are often employed in an attempt to manage violence and aggression. Whilst the enactment of violence may be interpreted to ‘cause’ this increase in service use, it is again important to consider the converse relationship; that violent behaviour is exacerbated by the upheaval associated with internal transfers, the frustration and boredom that occur as a result of longer stays of hospital, and the possible trauma that results from being detained (Antonysamy, 2013).

In order to improve treatment outcomes and save resources, an approach whereby a centralised team proactively reaches out to services users and their treatment teams may be beneficial. The recent NHS High Intensity User Service pioneered in Blackpool is an example of one such approach used in physical health services. The service’s primary aim is to reduce frequent use of A&E (Accident and Emergency) for service users who may gain more appropriate support elsewhere. The top 50 high intensity users of A&E are identified using data systems, and engagement is established to discuss current difficulties, and de-escalate social, emotional and financial stressors. After intervention, service users are discharged to the community and helped to manage relapse. Analysis of existing sites showed that three months post intervention, A&E attendances were down by 38%, whilst admissions were down by 51% (NHS, 2016). Total savings for the scheme were estimated at £2,757,380 over a 15-month period, resulting from an intervention aimed at just 100 service users (Johnston & Monteith, 2015). The small group of service users who disproportionately account for the majority of violent incidents may stand to benefit most from such specialised interventions, with additional benefits for other patients, staff and the NHS.

## Limitations

The main limitation of our analysis is that it is primarily descriptive in nature. The descriptions do not elucidate possible relationships between factors, nor does it elucidate causal mechanisms. Some measures of acute service use are likely to be directly caused by violence and aggression, such as transfers from acute adult wards to PICUs resulting from violent incidents. The relationship between violence/aggression and other acute service use metrics might be more complex. For example, the clear differences in OBDs amongst the cohorts could simply be because staying in hospital for longer creates more opportunity for incidents of violence or aggression to occur. Conversely, the reverse causal relationship could exist, with service users enacting violence/aggression being deemed too risky for discharge, thus accruing more OBDs. The direction of the relationship between these two factors is likely to vary amongst service users.

In addition, this study has this study has examined the enactment of violence and aggression within SLaM specifically. However, we believe that it will be, to some extent, generalizable across other healthcare settings, particularly within London, and particularly when considering the human and financial benefits of working with the small group of service users who enactment the vast majority of violence and aggression.

Finally, we were able to match 80.29% of service use and incident data, meaning that 19.71% of violent/aggressive incidents occurring during 2020 could not be linked to a service user with an inpatient stay during the same period. This is likely because the Trust-IDs used to identify individuals have been incorrectly entered into Datix, or have not been entered at all when recording incidents.

## Conclusion

Our results show that the distribution of incidents of violence and aggression across service users is heavily skewed, supporting the notion that a small proportion of service users disproportionately account for the majority of violent incidents on acute inpatient psychiatric wards. This suggests that providing targeted, personalised interventions to this cohort may have a hugely beneficial impact on total violence/aggression rates, in addition to improving individual care. Moreover, we have demonstrated that higher levels of violence and aggression enactment are associated with higher amounts of acute service use on acute adult wards and PICUs. This presents a strong economic argument for working closely with the most violent cohort of service users.

## Appendix A: Summary of violent/aggressive incidents enacted for low, medium, high and high and consistent violence cohorts combined (N = 1,858)

**Table:**

Category	% of all incidents	% of all incidents moderate or above	Sub-category	Percent of all incidents	% classed as moderate and above
Assault By patient including alleged	51.99	70.19			
			Actual Physical Assault By Patient On OTHER	1.51	71.43
			Actual Physical Assault By Patient On PATIENT	20.40	63.59
			Actual Physical Assault By Patient On STAFF	23.20	74.25
			Threatened Assault By Patient On OTHER	0.38	57.14
			Threatened assault By Patient On PATIENT	2.10	69.23
			Threatened Assault By Patient On STAFF	4.41	80.49
Challenging Behaviour	43.92	71.57			
			Actual Inappropriate Sexual Behaviour	0.48	88.89
			Aggression Resulting In Accidental Injury	1.24	65.22
			Alleged Inappropriate Sexual Behaviour	0.05	100.00
			Other/Not Stated	24.11	74.33
			Throwing Objects Aggressively	9.04	66.67
			Verbal Assault	8.99	68.86
Harassment by patient including alleged	2.74	50.98			
			Alleged Harassment By Patient On OTHER	0.05	100.00
			Alleged Harassment By Patient On PATIENT	0.75	85.71
			Alleged Harassment By Patient On STAFF	0.86	50.00
			Other Harassment By Patient On PATIENT	0.22	25.00
			Other Harassment By Patient On STAFF	0.48	33.33
			Racial Harassment By Patient On STAFF	0.16	0.00
			Sexual Harassment By Patient On PATIENT	0.05	100.00
			Sexual Harassment By Patient On STAFF	0.16	0.00
Sexual assault by patient including alleged	1.35	60.00			
			Actual Sexual Assault By Patient On PATIENT	0.16	100.00
			Actual Sexual Assault By Patient On STAFF	0.70	46.15
			Alleged Sexual Assault By Patient On OTHER	0.05	0.00
			Alleged Sexual Assault By Patient On PATIENT	0.32	83.33
			Alleged Sexual Assault By Patient On STAFF	0.11	50.00

## Appendix B: Summary of violent/aggressive incidents enacted for low violence cohort (N = 370)

**Table:**

Category	% of all incidents	% of all incidents moderate or above	Sub-category	% of all incidents	% classed as moderate and above
Assault By patient including alleged	43.51	65.22			
			Actual Physical Assault By Patient On OTHER	1.89	85.71
			Actual Physical Assault By Patient On PATIENT	17.57	52.31
			Actual Physical Assault By Patient On STAFF	20.81	74.03
			Threatened Assault By Patient On OTHER	0.00	0.00
			Threatened assault By Patient On PATIENT	1.35	40.00
			Threatened Assault By Patient On STAFF	1.89	85.71
Challenging Behaviour	51.35	61.58			
			Actual Inappropriate Sexual Behaviour	1.08	75.00
			Aggression Resulting In Accidental Injury	1.08	100.00
			Alleged Inappropriate Sexual Behaviour	0.00	0.00
			Other/Not Stated	25.41	61.70
			Throwing Objects Aggressively	13.78	56.86
			Verbal Assault	10.00	62.16
Harassment by patient including alleged	2.70	40.00			
			Alleged Harassment By Patient On OTHER	0.00	0.00
			Alleged Harassment By Patient On PATIENT	0.81	100.00
			Alleged Harassment By Patient On STAFF	1.08	25.00
			Other Harassment By Patient On PATIENT	0.27	0.00
			Other Harassment By Patient On STAFF	0.00	0.00
			Racial Harassment By Patient On STAFF	0.00	0.00
			Sexual Harassment By Patient On PATIENT	0.00	0.00
			Sexual Harassment By Patient On STAFF	0.54	0.00
Sexual assault by patient including alleged	2.43	77.78			
			Actual Sexual Assault By Patient On PATIENT	0.54	100.00
			Actual Sexual Assault By Patient On STAFF	0.81	66.67
			Alleged Sexual Assault By Patient On OTHER	0.00	0.00
			Alleged Sexual Assault By Patient On PATIENT	1.08	75.00
			Alleged Sexual Assault By Patient On STAFF	0.00	0.00

## Appendix C: Summary of violent/aggressive incidents enacted for medium violence cohort (N = 555)

**Table:**

Category	% of all incidents	% of all incidents moderate or above	Sub-category	% of all incidents	% classed as moderate and above
Assault By patient including alleged	51.35	65.96			
			Actual Physical Assault By Patient On OTHER	0.90	20.00
			Actual Physical Assault By Patient On PATIENT	23.42	65.38
			Actual Physical Assault By Patient On STAFF	20.54	67.54
			Threatened Assault By Patient On OTHER	0.36	0.00
			Threatened assault By Patient On PATIENT	1.98	63.64
			Threatened Assault By Patient On STAFF	4.14	78.26
Challenging Behaviour	45.59	73.91			
			Actual Inappropriate Sexual Behaviour	0.72	100.00
			Aggression Resulting In Accidental Injury	0.90	40.00
			Alleged Inappropriate Sexual Behaviour	0.00	0.00
			Other/Not Stated	24.86	79.71
			Throwing Objects Aggressively	10.45	65.52
			Verbal Assault	8.65	68.75
Harassment by patient including alleged (3.59%)	2.16	50.00			
			Alleged Harassment By Patient On OTHER	0.00	0.00
			Alleged Harassment By Patient On PATIENT	0.18	100.00
			Alleged Harassment By Patient On STAFF	0.54	66.67
			Other Harassment By Patient On PATIENT	0.36	0.00
			Other Harassment By Patient On STAFF	0.54	33.33
			Racial Harassment By Patient On STAFF	0.18	100.00
			Sexual Harassment By Patient On PATIENT	0.18	100.00
			Sexual Harassment By Patient On STAFF	0.18	0.00
Sexual assault by patient including alleged	0.90	80.00			
			Actual Sexual Assault By Patient On PATIENT	0.00	0.00
			Actual Sexual Assault By Patient On STAFF	0.72	75.00
			Alleged Sexual Assault By Patient On OTHER	0.00	0.00
			Alleged Sexual Assault By Patient On PATIENT	0.18	100.00
			Alleged Sexual Assault By Patient On STAFF	0.00	0.00

## Appendix D: Summary of violent/aggressive incidents enacted for high violence cohort (N = 598)

**Table:**

Category	% of all incidents	% of all incidents moderate or above	Sub-category	% of all incidents	% classed as moderate and above
Assault By patient including alleged	55.52	69.88			
			Actual Physical Assault By Patient On OTHER	1.67	80.00
			Actual Physical Assault By Patient On PATIENT	19.06	61.40
			Actual Physical Assault By Patient On STAFF	26.09	73.08
			Threatened Assault By Patient On OTHER	0.17	0.00
			Threatened assault By Patient On PATIENT	2.84	76.47
			Threatened Assault By Patient On STAFF	5.69	79.41
Challenging Behaviour	40.80	75.00			
			Actual Inappropriate Sexual Behaviour	0.17	100.00
			Aggression Resulting In Accidental Injury	1.67	50.00
			Alleged Inappropriate Sexual Behaviour	0.17	100.00
			Other/Not Stated	25.92	78.71
			Throwing Objects Aggressively	5.52	72.73
			Verbal Assault	7.36	68.18
Harassment by patient including alleged	2.17	46.15			
			Alleged Harassment By Patient On OTHER	0.00	0.00
			Alleged Harassment By Patient On PATIENT	0.33	50.00
			Alleged Harassment By Patient On STAFF	1.00	50.00
			Other Harassment By Patient On PATIENT	0.17	100.00
			Other Harassment By Patient On STAFF	0.50	33.33
			Racial Harassment By Patient On STAFF	0.17	0.00
			Sexual Harassment By Patient On PATIENT	0.00	0.00
			Sexual Harassment By Patient On STAFF	0.00	0.00
Sexual assault by patient including alleged	1.51	33.33			
			Actual Sexual Assault By Patient On PATIENT	0.17	100.00
			Actual Sexual Assault By Patient On STAFF	0.84	20.00
			Alleged Sexual Assault By Patient On OTHER	0.17	0.00
			Alleged Sexual Assault By Patient On PATIENT	0.00	0.00
			Alleged Sexual Assault By Patient On STAFF	0.33	50.00

## Appendix E: Summary of violent/aggressive incidents enacted for high and consistent violence cohort (incident N = 335)

**Table:**

Category	% of all incidents	% of all incidents moderate or above	Sub-category	% of all incidents	% classed as moderate and above
Assault By patient including alleged	56.12	80.85			
			Actual Physical Assault By Patient On OTHER	1.79	83.33
			Actual Physical Assault By Patient On PATIENT	20.90	74.29
			Actual Physical Assault By Patient On STAFF	25.07	85.71
			Threatened Assault By Patient On OTHER	1.19	75.00
			Threatened assault By Patient On PATIENT	1.79	83.33
			Threatened Assault By Patient On STAFF	5.37	83.33
Challenging Behaviour	38.51	75.19			
			Actual Inappropriate Sexual Behaviour	0.00	0.00
			Aggression Resulting In Accidental Injury	1.19	100.00
			Alleged Inappropriate Sexual Behaviour	0.00	0.00
			Other/Not Stated	18.21	70.49
			Throwing Objects Aggressively	7.76	80.77
			Verbal Assault	11.34	76.32
Harassment by patient including alleged	4.78	75.00			
			Alleged Harassment By Patient On OTHER	0.30	100.00
			Alleged Harassment By Patient On PATIENT	2.39	87.50
			Alleged Harassment By Patient On STAFF	0.90	66.67
			Other Harassment By Patient On PATIENT	0.00	0.00
			Other Harassment By Patient On STAFF	0.90	33.33
			Racial Harassment By Patient On STAFF	0.30	100.00
			Sexual Harassment By Patient On PATIENT	0.00	0.00
			Sexual Harassment By Patient On STAFF	0.00	0.00
Sexual assault by patient including alleged	0.60	50.00			
			Actual Sexual Assault By Patient On PATIENT	0.00	0.00
			Actual Sexual Assault By Patient On STAFF	0.30	0.00
			Alleged Sexual Assault By Patient On OTHER	0.00	0.00
			Alleged Sexual Assault By Patient On PATIENT	0.30	100.00
			Alleged Sexual Assault By Patient On STAFF	0.00	0.00

## Appendix F: Incident data available for each violent cohort

**Table:**

Violence Cohort	Incidents with data provided	Total incidents	Data completeness (%)
Low	370	453	81.68
Medium	555	676	82.10
High	598	714	83.75
High and consistent	335	415	80.72
Combined	1858	2258	82.29
